# TransPrimer: A Web-Based Tool for Automated Transcript-Specific RT-PCR/qPCR Primer Design Across Multiple Species

**DOI:** 10.21203/rs.3.rs-11225556/v1

**Published:** 2026-10-05

**Authors:** Junyeop Ahn, Yun H. Lee, Donald J Zack, Ping-Wu Zhang

**Affiliations:** 1. Department of Ophthalmology, Wilmer Eye Institute, Stem Cell Ocular Regenerative Medicine Center, Johns Hopkins University School of Medicine; Baltimore, Maryland, 21231, United States; 2. Vugle Health LLC, Casper, Wyoming, United States; 3. Department of Molecular Biology and Genetics, Department of Genetic Medicine, Solomon H. Snyder Department of Neuroscience, Center for Nanomedicine at the Wilmer Eye Institute, The Johns Hopkins University School of Medicine, Baltimore, Maryland, United States

**Keywords:** transcript-specific PCR, pan-transcript amplification, RNA variant, non-coding RNA, exon junction, primer design, web server tool, alternative splicing, quantitative PCR, RefSeq

## Abstract

Transcript-specific RT-PCR/qPCR primer design is challenging when closely related RNA variants share extensive exonic sequence and differ primarily in exon connectivity. We developed TransPrimer, a web application for automated transcript-specific and pan-transcript primer design. TransPrimer retrieves curated RefSeq protein-coding and non-coding transcripts for six species and compares sibling transcripts to identify discriminating exons and exon–exon junctions (EEJs). The application automatically determines whether transcript specificity can be achieved using exon-targeted primers or requires single- or dual-EEJ strategies. A complementary pan-transcript mode identifies primer pairs that amplify all annotated transcripts of a gene, when feasible, or the largest compatible subset. Junction-spanning primers are evaluated using an arm-specific thermodynamic framework that independently calculates melting temperatures (Tm) for the full primer and its two exon-matching arms using nearest-neighbor thermodynamics and mixed-salt correction. Primer3-based criteria are integrated for partner-primer design and evaluation. User-adjustable reaction parameters, interactive transcript visualization, and custom sequence input support flexible assay development, including novel or externally annotated transcripts. By integrating transcript retrieval, structural comparison, strategy selection, thermodynamic evaluation, and primer generation, TransPrimer reduces manual transcript inspection and facilitates transcript-specific and gene-level RT-PCR/qPCR assay development across diverse transcript architectures.URL: https://transprimer.vercel.app/

## Introduction

Alternative splicing generates transcript diversity through mechanisms such as alternative exon usage, exon skipping, alternative splice-site selection, and intron retention [1–3]. Closely related transcript variants can share most or all exon-internal sequence while differing in the connectivity of those exons. This creates a practical challenge for targeted RT-PCR and qPCR, because primers placed entirely within shared exons may amplify multiple sibling transcripts and obscure variant-specific measurements [4]. The same design problem can arise for protein-coding mRNAs and non-coding RNAs and across diverse eukaryotic species in which genes produce multiple annotated transcript variants.

High-throughput short- and long-read RNA sequencing can resolve transcript diversity at scale, but targeted PCR remains widely used for validation and focused quantification because RNA sequencing is time-consuming and costly [5–8]. Exon-exon junctions provide an additional source of specificity because a junction encodes the adjacency of two exon sequences in mature RNA. A primer that spans an informative junction can therefore distinguish transcripts even when neither exon contains a sufficiently large unique internal region.

Several tools support isoform-aware or exon-junction primer design. “ExonSurfer” integrates automatic junction selection, transcript/off-target assessment, SNP avoidance, and Primer3-based design in a web workflow [9]. “Ex-Ex Primer” was developed specifically for oligonucleotides spanning spliced regions [10], whereas “Primer-BLAST” combines primer generation with broad specificity searches and permits junction-spanning constraints [11]. These resources demonstrate the value of transcript-aware primer design, while also using different transcript databases, junction rules, and optimization strategies. “ExonSurfer” provides input from Ensembl only; “Ex-Ex Primer” requires users to manually select primer-design locations, whereas “Primer-BLAST” requires users to provide more detailed sequence information and design parameters before initiating primer design.

We developed TransPrimer, an automated, rapid, and optimized web-based platform that addresses a key decision preceding primer optimization: identifying the sequence features that can discriminate a selected transcript from closely related isoforms, or, alternatively, the shared regions suitable for amplification across an entire transcript set. TransPrimer automatically compares sibling RefSeq transcripts, identifies transcript-discriminating exon-internal regions and exon–exon junctions (EEJs), and applies a Tm-guided EEJ design strategy when junction-specific recognition is required [12]. The platform also supports pan-transcript primer design for gene-level assays and provides a custom-transcript workflow for novel, user-defined, or otherwise unavailable transcript models. Custom inputs are therefore not restricted to NCBI-accessioned transcripts; full-length sequences obtained from external databases or novel transcript discovery can be incorporated into the same comparison and design framework, using coordinate-aware alignment when RefSeq annotations are available and sequence-derived relative block alignment otherwise. Here, we describe the software architecture, multi-species transcript-retrieval workflow, transcript-comparison and primer-selection algorithms, thermodynamic implementation, user interface, and representative computational evaluation of TransPrimer.

## Implementation

### Programming language and software packages

The computational engine is implemented in Python using the FastAPI framework, and the user interface is developed with React, TypeScript, and Vite. Primer candidate generation and oligonucleotide quality assessment are supported by Primer3 through the primer3-py library [13]. Primer3-derived parameters used during candidate evaluation include primer length, GC content, predicted hairpin formation, self-dimerization, cross-dimerization, and amplicon size.

### Transcript data sources and retrieval

Transcript information is obtained from the NCBI Reference Sequence (RefSeq) database [14]. The current web application supports six species: Homo sapiens, Mus musculus, Danio rerio, Rattus norvegicus, Drosophila melanogaster, and Saccharomyces cerevisiae. Curated protein-coding mRNA transcripts (NM accessions) and curated non-coding RNA transcripts (NR accessions), where available, are included in the searchable transcript set. For each analyzed gene, the application retrieves exon coordinates and full-length transcript sequences for the selected transcript and its sibling curated variants. Records are obtained on demand through NCBI dataset and sequence-retrieval services and are cached locally when available to reduce repeated external requests while preserving a responsive interactive workflow. Because the application is no longer restricted to protein-coding genes, transcript comparison and primer design can be performed for genes represented by curated coding transcripts, curated non-coding transcripts, or both. The Custom Sequence module does not directly retrieve external transcript databases; instead, users may paste a full-length transcript sequence with optional exon location indications, allowing the sequence to be analyzed together with RefSeq records or with other user-supplied transcripts.

### Transcript comparison and target-site classification

The workflow begins with selection of a supported species followed by a gene symbol or curated RefSeq transcript accession. A gene-symbol query returns the available curated NM and NR transcripts for transcript selection. A direct accession query is resolved to its parent gene and triggers retrieval of the corresponding sibling transcript set. The selected transcript is then evaluated at both exon-structure and nucleotide-sequence levels. This species-aware resolution step allows the same downstream design logic to be applied across the supported vertebrate, invertebrate, and yeast transcriptomes.

At the sequence level, windows from the target transcript are compared with sibling curated transcript sequences. A sequence window is considered target-discriminating when the identical sequence is present in the target transcript and absent from all compared sibling transcripts. Candidate regions located entirely within an exon can support EEJ-independent primer placement, whereas target-discriminating sequences that span an exon boundary are treated as candidate EEJ sites. When no single feature is sufficient, combinations of two transcript features can be evaluated, including exon pairs, an exon plus an EEJ, or two EEJs. This permits the workflow to represent single-EEJ and dual-EEJ primer strategies rather than assuming that one junction primer is always sufficient. In a complementary pan-transcript workflow, the analysis is reversed: instead of searching for target-discriminating sequence, the program searches for primer-binding regions shared across the gene’s annotated transcript set and attempts to identify a pair that amplifies all transcripts. When no pair can cover every transcript under the active design constraints, the tool seeks a solution covering the largest feasible subset and reports the transcript coverage of the proposed design.

In Custom Sequence mode, full-length sequences are first normalized and aligned into a common transcript-comparison representation. When a custom transcript is analyzed together with coordinate-resolved RefSeq models, shared sequence regions are used to place the custom sequence relative to the existing exon-coordinate framework. If only coordinate-free custom transcripts are compared, users may provide exon boundaries as nucleotide positions or exon lengths; however, exon specification is optional because the program can infer relative shared and transcript-specific sequence blocks from sequence correspondence and use those blocks as a common structure. These inferred blocks support junction and region comparisons without assuming that user exon numbers correspond across transcripts. The resulting representation is then passed to the same strategy-selection logic used for RefSeq analyses.

### Tm-guided exon-exon junction primer design

For a junction-spanning candidate, the complete primer and the two exon-matching arms are evaluated separately. Full-length oligonucleotide Tm is calculated using the SantaLucia nearest-neighbor model [15] with the Owczarzy mixed monovalent/divalent salt correction [16]. User-adjustable inputs include monovalent salt, Mg2+, dNTP, and primer concentrations. Short arm segments below 10 nucleotides are evaluated with the Wallace approximation [17]. Under the default design settings, the full junction primer is selected near 60–65 °C, while each exon-matching arm is constrained relative to the selected annealing condition. The current default uses an approximately 15 °C arm-specific margin, allowing the software to favor primers whose full-length junction match is stable while either partial exon match alone is comparatively unfavorable.

### Primer-pair generation and reporting

After an EEJ primer or exon-internal candidate is selected, the program searches for compatible partner primers and reports primer sequence, predicted Tm, Tm difference relative to the junction primer when applicable, GC content, primer length, expected amplicon size, transcript coordinates, and the transcript set expected to be amplified. Candidate evaluation uses Primer3-derived oligonucleotide quality metrics in addition to the transcript-discrimination or pan-transcript coverage logic. The application is intended to identify transcript-aware candidate pairs; sequence specificity is evaluated primarily in relation to the transcript set under analysis, so complementary genome-wide off-target assessment remains advisable before experimental use.

### Web interface, pan-transcript mode, and custom transcript input

The browser interface combines species selection, transcript structure visualization, transcript-specific or pan-transcript strategy selection, and primer design. Exon models display sibling isoforms and highlight candidate discriminating or shared regions, allowing users to see why a transcript is classified as compatible with exon-targeted, single-EEJ, dual-EEJ, or currently infeasible amplification. For a selected junction, an interactive designer displays the local transcript sequence and allows the user to adjust the lengths of the 5’ donor-exon and 3’ acceptor-exon arms while viewing full-primer and arm-specific Tm values. The pan-transcript mode instead evaluates sequence regions shared across the selected gene’s transcript set and searches for primer pairs that amplify all annotated transcripts when possible, or the largest feasible subset when a universal pair cannot satisfy the design constraints. A custom-sequence workflow extends the application beyond the transcripts currently retrievable from RefSeq. Users can provide transcript and exon sequence information for novel, unannotated, non-accessioned, or otherwise unavailable transcript models, after which the same transcript-comparison logic can be applied to design transcript-specific primers either within distinguishing exon sequence or across informative EEJs. This allows user-defined transcript sets to be analyzed without requiring an existing NCBI accession.

The Custom sequence tab accepts complete 5′→3′ transcript sequences. Each entry can be designated as a target or as a transcript to avoid, and custom entries can be mixed with Existing RefSeq gene/transcript entries. Exon boundaries may be entered as positions or lengths but are not required. After Compare & design, the interface displays custom and existing models on a shared relative block map, enabling transcript-specific or shared-amplification/co-amplification design from the harmonized structures. Custom sequences then use the same downstream primer-design functions as database-derived transcripts, including EEJ-dependent and EEJ-independent candidates, single- or dual-EEJ strategies where applicable, partner-primer generation, and user-adjustable monovalent salt, Mg2+, dNTP, and primer concentration settings.

## Results

### Integrated transcript-specific design workflow

The interface links species-aware transcript browsing, transcript-discrimination analysis, pan-transcript analysis, and primer design in a single session (Figure 1). After the user selects a transcript, sibling exon structures are displayed on a common coordinate system and candidate target features are marked directly on the models. The tool can therefore show whether a transcript contains an exon-internal discriminating region or whether discrimination depends on exon connectivity. Users may instead choose the pan-transcript objective to identify common primer-binding regions across the gene’s annotated transcript set. In the GAPDH example shown in Figure 1, the transcript display is followed by a strategy map that distinguishes EEJ-independent, EEJ-dependent, and infeasible regions. Selecting an EEJ opens the junction designer, where the two exon-matching arms can be adjusted and evaluated independently before compatible partner primers are displayed. The same core workflow is available for curated non-coding transcripts and for supported non-human species, while custom transcript input provides an annotation-independent route for user-defined transcript sets. The expanded Custom Sequence workflow extends this same decision process to user-supplied transcript models and is illustrated in Figure 4.

### Representative comparison with existing primer-design tools

We evaluated automated transcript-specific design across representative human transcript sets using the default workflows shown in the original comparison, without manually specifying a priming site or adjusting design parameters. The comparison included Primer-BLAST and ExonSurfer, with additional qualitative observations from Ex-Ex Primer. Because these tools use different transcript databases, design constraints, and optimization objectives, the comparison is intended to illustrate workflow coverage rather than provide a standardized performance ranking. This benchmark predates the current expansion of TransPrimer to additional species, curated non-coding RNAs, pan-transcript design, and full custom transcript input; those capabilities therefore are not represented in Figure 3. The current custom-sequence interface and coordinate-independent alignment workflow are illustrated separately in Figure 4.

Across the 35 RefSeq transcript cases displayed for GAPDH, OPA1, ERBB4, and TCF7L2 in Figure 3, TransPrimer returned a candidate transcript-specific design for 29 cases, whereas Primer-BLAST returned a design for 17 under the tested settings. Several of the additional TransPrimer solutions used dual-EEJ strategies, illustrating the benefit of evaluating combinations of transcript features when no single unique exon or junction is sufficient. Some transcripts remained classified as infeasible under the tested constraints, which is preferable to forcing a nominally specific primer pair when the transcript structure does not provide an adequate discriminating feature.

ExonSurfer was additionally examined using corresponding Ensembl transcript identifiers for GAPDH. In the displayed panel, automated designs were returned for two of six ENST transcripts under the tested settings. This observation should be interpreted cautiously because ExonSurfer is built around Ensembl transcript models and its optimization goals differ from the RefSeq-centered workflow described here. Ex-Ex Primer was also examined during development; in several cases it required manual junction selection or produced high-Tm candidates. These comparisons reinforce that the primary contribution of TransPrimer is not a universal replacement for existing tools, but an automated sibling-transcript decision framework that can search exon-targeted, single-EEJ, and dual-EEJ solutions within the same workflow.

### Custom-sequence integration and coordinate-independent transcript alignment

The Custom Sequence workflow extends transcript-aware primer design to full-length transcript models that are not directly retrieved through the integrated RefSeq search (Figure 4A). Users can paste a novel transcript sequence or a sequence corresponding to an external annotation model, such as an Ensembl transcript, and analyze it alone, with additional custom transcripts, or together with Existing RefSeq transcripts. In the example shown, ENST00000708787.1 is supplied as the target and compared with curated APEX1 RefSeq transcripts designated for avoidance.

Custom inputs do not require preassigned genomic coordinates. When coordinate-resolved RefSeq transcripts are present, the application uses sequence correspondence to align the user-supplied transcript to the existing coordinate-derived structure. When only coordinate-free custom sequences are compared, exon boundaries may be supplied as positions or lengths, but this annotation is optional: shared-region sequence analysis can derive relative shared and transcript-specific blocks and create inferred exon/block assignments for a common comparison structure (Figure 4B). The aligned models are then processed by the same transcript-specific or shared-amplification workflow used elsewhere in the application, including EEJ-dependent and EEJ-independent primer selection, single- or dual-EEJ strategies where feasible, partner-primer generation, and the same user-adjustable thermodynamic and buffer-condition settings.

## Discussion

TransPrimer was developed as an automated, rapid, and optimized web-based platform for transcript-specific RT-PCR/qPCR primer design. A central feature of the platform is that it addresses not only primer optimization but also the upstream decision of where transcript-discriminating primers should be placed. Alternative splicing generates extensive transcript diversity, with related isoforms often sharing most of their exonic sequences while differing primarily in exon composition or connectivity, creating challenges for transcript-specific amplification [3]. Rather than requiring users to manually inspect transcript structures and select candidate primer locations, TransPrimer automatically compares related transcript isoforms, identifies informative exon-internal regions and exon–exon junctions (EEJs), and selects suitable regions for primer design according to the structural and sequence relationships among transcripts. When EEJ recognition is required, the platform further incorporates a Tm-guided design strategy that evaluates the thermodynamic contribution of each junction-spanning arm. Together, these functions integrate transcript comparison, primer-location selection, and thermodynamic optimization into a unified workflow.

The current annotation-based workflow uses curated NCBI RefSeq transcript models from six supported species and includes both NM protein-coding mRNAs and NR non-coding RNAs where available. RefSeq provides a curated and relatively stable collection of genomic, transcript, and protein reference sequences supported by computational annotation, manual curation, and available biological evidence [14]. Nevertheless, the complexity of alternative transcription and splicing means that no single annotation resource necessarily captures every predicted, tissue-specific, disease-associated, strain-specific, or experimentally observed transcript isoform [3, 14]. Accordingly, a transcript classified by TransPrimer as distinguishable or indistinguishable should be interpreted in the context of the transcript set included in the analysis, because additional isoforms outside the retrieved RefSeq set may alter these relationships.

To mitigate this limitation, TransPrimer provides a custom-transcript workflow that allows users to directly input full-length sequences for novel, externally annotated, or otherwise unavailable transcript models. The platform is therefore not restricted to NCBI-accessioned transcripts and can accommodate sequences obtained from other annotation resources or experimental transcript-discovery approaches. User-provided exon boundaries can further refine these comparisons but are optional. When genomic coordinates or exon boundaries are unavailable, TransPrimer derives relative sequence blocks from shared-region sequence analysis to facilitate transcript comparison. These inferred blocks should be interpreted as relative comparison structures for primer-design purposes rather than independently validated genomic exon annotations. Consequently, the reliability of custom-mode analysis depends on the completeness and accuracy of the transcript sequences supplied by the user.

A related limitation is that the current annotation-based workflow does not directly retrieve Ensembl transcript models. Because transcript databases differ in annotation strategies, curation criteria, and transcript coverage, some isoforms represented in Ensembl or other resources may not be included in the curated RefSeq dataset [3, 14]. Users interested in these transcripts can currently analyze them through the custom-transcript workflow by supplying their full-length sequences directly. Future versions of TransPrimer could integrate additional transcript annotation resources, including Ensembl, to expand transcript coverage and enable direct cross-database transcript comparison.

Sequence-level specificity in TransPrimer is evaluated primarily among sibling or user-defined transcripts included in the active analysis and therefore should not be interpreted as a comprehensive genome- or transcriptome-wide off-target assessment. Primer-BLAST, for example, combines primer design with database searches to identify potential unintended amplification targets, illustrating the importance of evaluating primer specificity beyond the intended transcript set [11]. Complementary genome- or transcriptome-wide specificity analysis is therefore recommended before experimental application, particularly for highly quantitative or diagnostic assays. Some transcript relationships also impose intrinsic limitations that cannot be overcome by primer-design algorithms alone. Transcripts sharing identical informative exon connectivity may remain indistinguishable by junction-specific PCR. Similarly, for pan-transcript assays, a universal primer pair may not exist when no sufficiently conserved primer-binding regions satisfy the selected thermodynamic, amplicon-size, and oligonucleotide-quality constraints. In such cases, TransPrimer reports a design covering the largest feasible transcript subset rather than implying complete transcript coverage.

Primer performance also depends on experimental variables beyond sequence selection, including polymerase chemistry, buffer composition, ionic conditions, annealing temperature, template abundance, secondary structure, mismatch position, and local sequence context [18]. The arm-specific thermodynamic criterion implemented in TransPrimer should therefore be considered a rational design constraint rather than an absolute predictor of amplification behavior. Experimental validation and optimization of reaction conditions remain important when establishing final RT-PCR or qPCR assays.

The present comparison of TransPrimer with existing exon-junction and transcript-aware primer-design tools represents an evaluation of workflow and functionality rather than a fully standardized performance benchmark. Existing tools differ in reference transcript sources, search spaces, default parameters, specificity algorithms, levels of automation, and intended applications, making direct quantitative comparisons challenging. Future studies using harmonized transcript datasets, predefined design criteria, standardized specificity metrics, and experimental validation across a broader range of genes and transcript architectures will enable more rigorous assessment of relative performance

## Conclusion

TransPrimer provides an automated, multi-species, transcript-aware web platform that integrates transcript structure analysis with RT-PCR/qPCR primer design. By combining on-demand RefSeq retrieval, automated sibling-transcript comparison, exon- and EEJ-based target identification, Tm-guided junction-primer design, pan-transcript analysis, Primer3-supported optimization, and flexible custom-transcript input, TransPrimer converts a multi-step manual process into an integrated and reproducible computational workflow. Importantly, the platform extends conventional primer-design workflows beyond primer optimization by automatically identifying transcript-discriminating sequence features and determining appropriate design strategies before primer optimization. Support for protein-coding and non-coding transcripts across six species, together with annotation-independent custom transcript analysis, further broadens its applicability to diverse transcriptomic studies. Future integration of additional transcript annotation resources and more comprehensive genome- and transcriptome-wide specificity analysis should further expand the utility of TransPrimer as transcript annotations continue to increase in complexity.

## Figures and Tables

**Figure 1.**
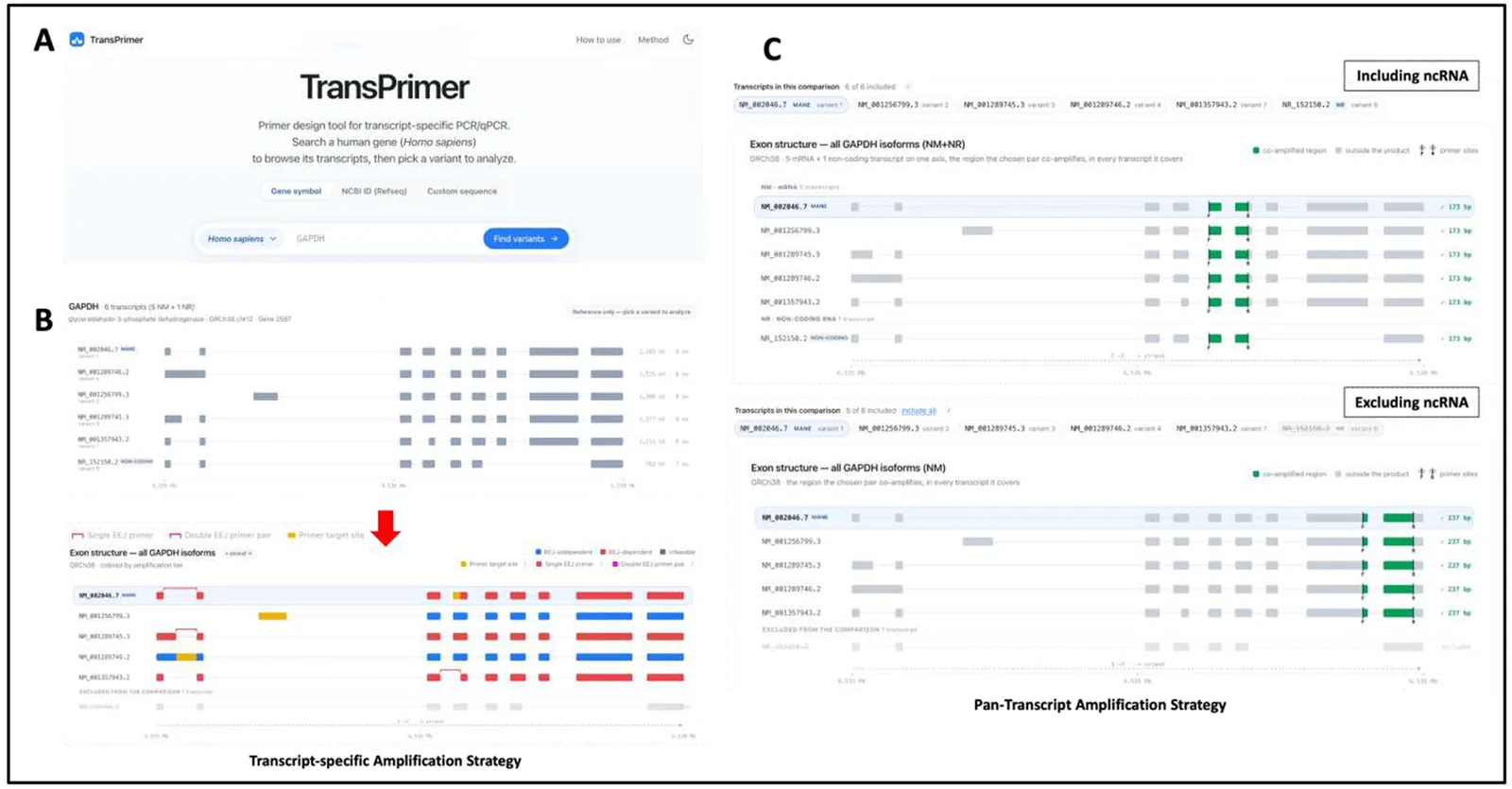
Automated transcript comparison, primer-design strategy selection, and specificity assessment using TransPrimer. (A) TransPrimer input interface and transcript-structure visualization. Users select a species and gene or provide a RefSeq accession or custom sequence, after which the application retrieves and displays available curated RefSeq transcripts for structural comparison. (B) Automated identification and classification of candidate primer-design regions. Based on differences among transcript structures, the application identifies regions suitable for single-EEJ, double-EEJ, exon-targeted, or shared primer designs, enabling automated selection of transcript-discriminating strategies. (C) Multiple transcript-level specificity assessment of a designed primer pair using GAPDH as an example. The predicted amplicon is mapped across the compared transcripts to identify potential co-amplification. Users can compare all available transcripts, including non-coding RNAs (top panel), or restrict the analysis to protein-coding mRNA transcripts (bottom panel). Green regions indicate predicted amplified regions, while primer positions and regions outside the predicted amplicon provide structural context

**Figure 2.**
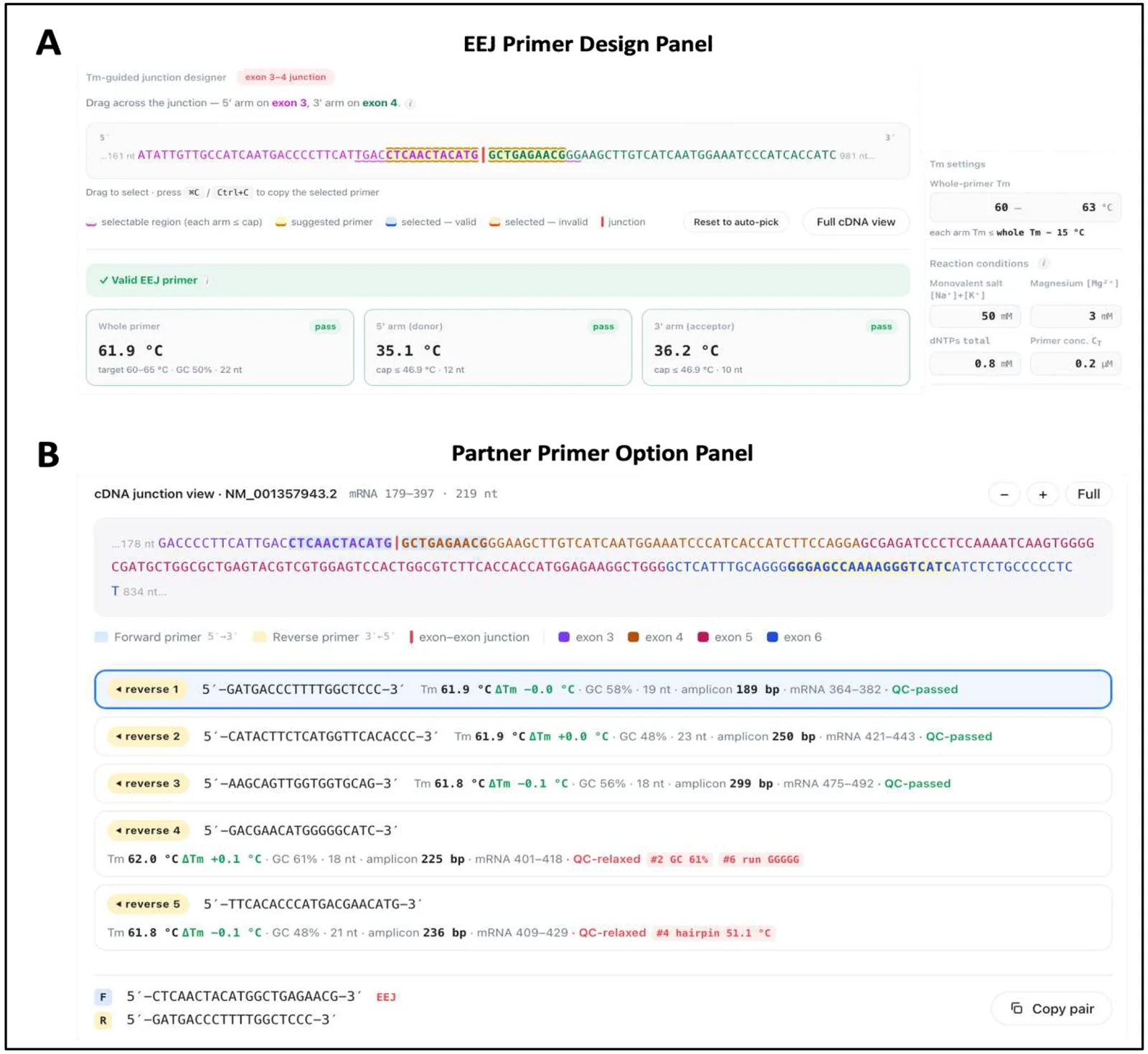
Tm-guided exon–exon junction primer design and quality-control interface. (A) Interactive Tm-guided exon–exon junction (EEJ) primer designer. Users can adjust primer placement across a selected EEJ while the application calculates the whole-primer Tm and the Tm values of the 5′ donor-exon and 3′ acceptor-exon arms independently. Primer validity is evaluated using the user-defined annealing temperature and arm-specific Tm criterion, together with primer length and GC content. Reaction conditions, including monovalent ion, Mg^2+^, dNTP, and primer concentrations, can be adjusted for Tm calculation. (B) Automated candidate primer-pair design and quality-control evaluation. Candidate primers are displayed within the transcript-specific cDNA sequence with calculated Tm, GC content, amplicon size, and quality-control metrics. Primer candidates meeting the design criteria are labeled QC-passed, whereas potential issues, including excessive GC content, homopolymer runs, and predicted hairpin formation, are flagged to facilitate selection of an optimized primer pair

**Figure 3.**
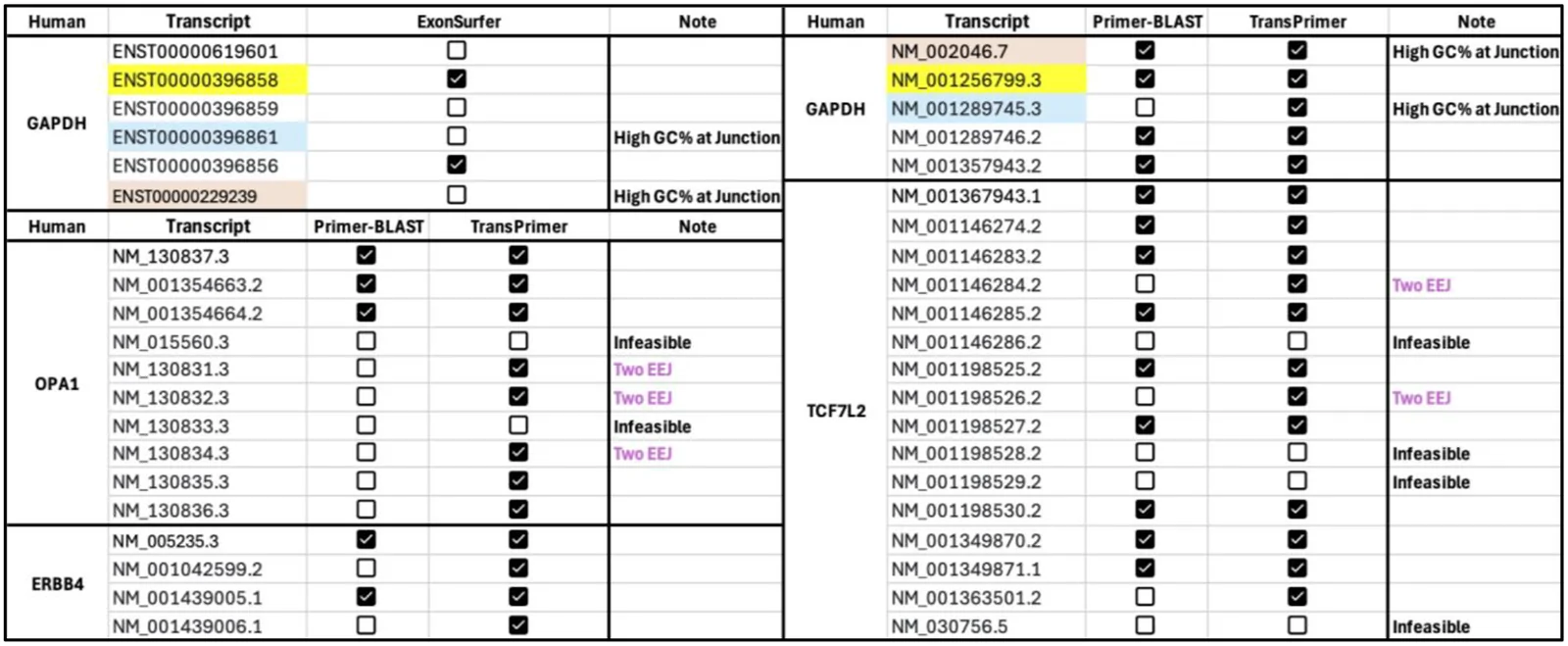
Representative automated transcript-specific primer-design comparison. Checked boxes indicate that a candidate design was generated under the tested default conditions. RefSeq transcript cases for GAPDH, OPA1, ERBB4, and TCF7L2 compare Primer-BLAST with TransPrimer; the separate GAPDH panel uses corresponding Ensembl transcript identifiers for ExonSurfer. Notes identify cases requiring dual-EEJ designs, cases classified as infeasible, and junctions with challenging GC content. Differences in transcript databases and tool objectives limit direct numerical comparison

**Figure 4.**
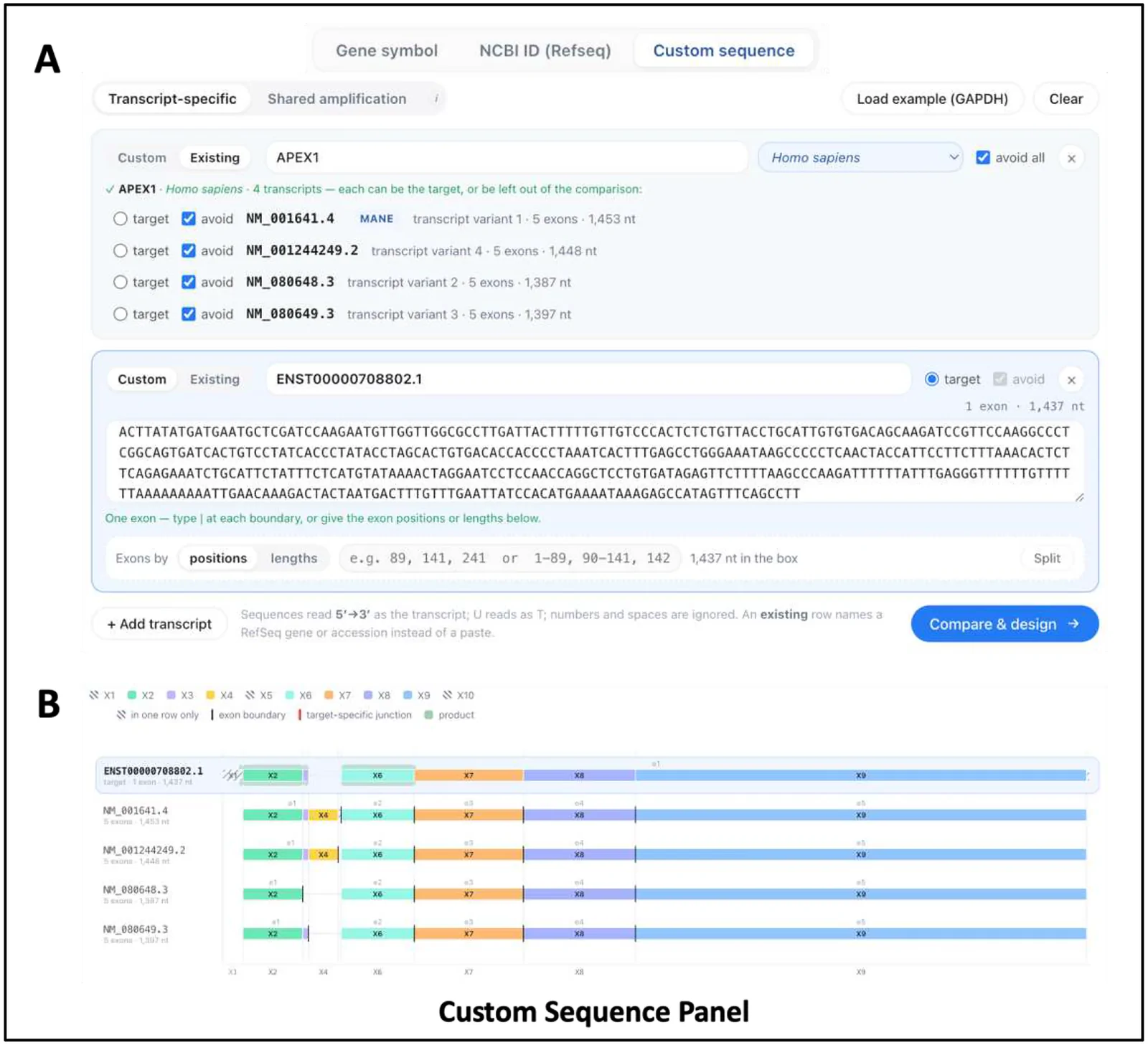
Custom-sequence integration and coordinate-independent transcript alignment. (A) The Custom sequence interface accepts complete transcript sequences as target or comparison/avoid entries and permits custom transcripts to be combined with Existing RefSeq gene/transcript entries. The example shows a user-supplied Ensembl transcript sequence (ENST00000708787.1) as the target and APEX1 RefSeq transcripts as comparison sequences. Exon boundaries can be entered as positions or lengths but are optional. (B) The comparison view projects the custom target and RefSeq transcripts onto a shared relative sequence-block framework. When coordinate-resolved RefSeq structures are available, they provide the coordinate framework; for coordinate-free custom-only comparisons, user-supplied exon boundaries or shared-region sequence analysis can define relative exon/block relationships. This harmonized representation supports the same transcript-specific and shared-amplification primer-design functions, including EEJ design and user-defined reaction conditions

**Table 1.** End-to-end workflow implemented by TransPrimer.

Stage	Main operation	Primary output
**Input Resolution**	Select species; resolve gene symbol or curated RefSeq accession; retrieve sibling NM/NR transcripts, or load user-defined transcript information.	Target transcript set
**Structure Comparison**	Compare exon coordinates and transcript organization across sibling or custom transcript models.	Candidate distinguishing/shared exons and junctions
**Sequence Comparison**	Compare target transcript sequence with sibling transcripts, or search for sequence conserved across the transcript set.	Transcript-specific or pan-transcript sequence features
**Strategy Selection**	Classify feasible exon-targeted, single-EEJ, exon-pair, dual-EEJ, or pan-transcript designs.	Amplification strategy and expected transcript coverage
**EEJ Thermodynamics**	Evaluate full junction primer and 5’/3’ arms using user-specified ionic conditions.	Valid/invalid EEJ candidates
**Partner Design**	Generate compatible partner primers and apply Primer3 quality criteria.	Ranked primer pairs
**Visualization**	Display transcript structures, distinguishing/shared regions, junctions, local sequence, primer metrics, and transcript coverage.	Interactive design report

## Data Availability

TransPrimer is publicly available at https://tm-junction-web.vercel.app/. The source code is available under the MIT License at https://github.com/leeonthe/tm-junction-web. The application accesses curated RefSeq transcript annotation data from NCBI through an on-demand, cache-assisted retrieval workflow and currently supports Homo sapiens, Mus musculus, Danio rerio, Rattus norvegicus, Drosophila melanogaster, and Saccharomyces cerevisiae. Both curated protein-coding mRNAs and curated non-coding RNAs can be analyzed where available. The application also supports user-defined transcript input for transcripts that are novel, unannotated, non-accessioned, or otherwise unavailable through the integrated RefSeq search. Data used for the representative transcript-design comparisons are summarized in Figure 3 and can be provided as supplementary material with the final submission.
